# Cysteine 904 Is Required for Maximal Insulin Degrading Enzyme Activity and Polyanion Activation

**DOI:** 10.1371/journal.pone.0046790

**Published:** 2012-10-15

**Authors:** Eun Suk Song, Manana Melikishvili, Michael G. Fried, Maria A. Juliano, Luiz Juliano, David W. Rodgers, Louis B. Hersh

**Affiliations:** 1 Department of Molecular and Cellular Biochemistry and the Center for Structural Biology, University of Kentucky, Lexington, Kentucky, United States of America; 2 Department of Biophysics, Universidade Federal de Sao Paulo, Escola Paulista de Medicina, Rua Tres de Maio, Sao Paulo, Brazil; Omaha Veterans Affairs Medical Center, United States of America

## Abstract

Cysteine residues in insulin degrading enzyme have been reported as non-critical for its activity. We found that converting the twelve cysteine residues in rat insulin degrading enzyme (IDE) to serines resulted in a cysteine-free form of the enzyme with reduced activity and decreased activation by polyanions. Mutation of each cysteine residue individually revealed cysteine 904 as the key residue required for maximal activity and polyanion activation, although other cysteines affect polyanion binding to a lesser extent. Based on the structure of IDE, Asn 575 was identified as a potential hydrogen bond partner for Cys904 and mutation of this residue also reduced activity and decreased polyanion activation. The oligomerization state of IDE did not correlate with its activity, with the dimer being the predominant form in all the samples examined. These data suggest that there are several conformational states of the dimer that affect activity and polyanion activation.

## Introduction

Insulin degrading enzyme (IDE), a zinc metalloprotease of the M16A family (EC 3.4.24.56), is involved in a number of important physiological processes. Most prominent are the enzyme's roles in type 2 diabetes [1] and Alzheimer's disease [2]–[4]. IDE also has been implicated in regulating cellular ubiquitin levels [5], natriuretic peptide-mediated signaling [6], and as a receptor for Varicella-Zoster Virus [7].

There have been several recent structures of IDE [8]–[11] or cysteine-free IDE in which serine residues replace cysteine residues [12]–[14] either alone or in complex with substrates [8]–[14]. These structures show that IDE is composed of four structurally similar domains with two each in the N- and C-terminal halves of the molecule (IDE-N and IDE-C) [10], [11]. In the structures obtained to date, the enzyme exists in a closed conformation in which the two halves of the molecule interact extensively and in which bound substrates are trapped within a large enclosed cavity. In order for catalysis to occur the enzyme must adopt an open conformation to bind substrates and to release products, much like a clam opening and closing. Although no IDE structure in the open conformation has been reported, insight into the open conformation comes from the related bacterial enzyme pitrilysin whose structure has been solved in an open form (Protein Data Bank entry 1Q2L).

Tang and coworkers showed that disrupting several hydrogen bonds that were presumed to help stabilize the closed conformation increased the rate of hydrolysis by IDE suggesting that the transition from the closed to open conformation is rate-limiting [10]. However, the finding that different substrates exhibit different kcat values requires that either substrate/product identity affects the rate of opening or that substrate cleavage in itself is rate limiting. This follows since if the conformational change that causes opening of the enzyme were solely rate limiting and substrate-independent, all IDE substrates would exhibit the same kcat, and this is clearly not the case [15].

In addition to the catalytic site, IDE contains a distal or exo site, which can bind the distal portion of extended substrates [9], [10]. Small peptides can also bind to this site, allosterically activating IDE [9]. Additionally there is an activation site on the inner surface of the C-terminal half of the molecule that binds polyanions including ATP [8]. With small synthetic substrates, occupation of this polyanion site increases rates fifty to a hundred fold [16].

Several studies have used the cysteine-free form of IDE to study aspects of IDE biology [12]–[15], [17]. When we generated rat cysteine-free IDE we found that catalytic activity was decreased and that the ability to activate the enzyme by polyanions was compromised. We report here a study on the role of cysteine residues in the activity and polyanion activation of IDE and identify cysteine 904 and its probable interacting partner asparagine 575 as key residues required for maximal IDE activity and polyanion activation.

## Materials and Methods

### Materials

Tris(2-carboxyethyl)phosphine (TCEP) was obtained from Hampton Research Corp. Abz-GGFLRKHGQ-EDDnp was prepared as previously described [18]. A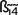 was purchased from AnaSpec, while insulin was from Sigma Chem. Co.

### Preparation of IDE and its mutant variants

IDE and its mutant forms were expressed in 200 ml cultures of Sf9 insect cells as previously described [8]. The enzyme was purified by HIS-select Ni-NTA agarose (Sigma) affinity chromatography. Typically enzyme of greater than 90% purity, as judged by Coomassie staining of SDS-PAGE gels, was obtained, see Figure S1 for representative gels. Mutations were introduced into IDE using the QuickChange mutagenesis kit (Stratagene). For generating cysteine-free IDE we made cysteine substitutions in three cassettes in which individual cysteines were mutated to serines and then the mutated portions of the cDNAs were cloned back into the pFast Bac expression vector using appropriate restriction sites. We also generated single point mutants in which each of the 12 cysteines were converted to serines or where Asn575 was changed to Leu or Thr, or Cys 904 was changed to Ala. In each case we used pFast Bac HTb-IDE as the template. All mutations were confirmed by DNA sequencing (Eurofins MWG Operon).

### Enzyme Assays

**T**he fluorogenic peptide Abz-GGFLRKHGQ-EDDnp at 10 µM in 50 mM Tris-HCl buffer, pH 7.4 was used for routine measurements of IDE activity as previously described [16]. Cleavage of this peptide by IDE occurs at the RK bond relieving fluorescence quenching between the aminobenzyl (Abz) and dinitrophenyl (EDDnp) groups. The reaction was monitored continuously using a SpectraMax Gemini XS fluorescence plate reader at an excitation of 318 nm and an emission of 419 nm. Graphpad software was used to fit kinetic data to either a hyperbolic substrate versus velocity response curve or to a sigmoidal response curve.

With A
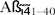
_1–40_ or insulin as substrate the reaction was measured by following the disappearance of the substrate peak by HPLC. Reaction mixtures containing 50 mM Tris-HCl buffer, pH 7.4, 10 µM A
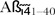
_1–40_ or 10 µM insulin and enzyme in a total volume of 100 µl were incubated at 37°C for 0.5 or 1 hr. The reaction was stopped by the addition of trifluoroacetic acid (TFA) to a final concentration of 0.5%. The quenched reaction was applied to a Vydac C_4_ HPLC column and an acetonitrile gradient developed as previously described [16].

### Analytical ultracentrifugation

Sedimentation velocity measurements were made at 4°C and 40,000 rpm, in a Beckman XL-A analytical ultracentrifuge (Beckman, Fullerton, CA) with an An-60 Ti rotor. The sample buffer contained 50 mM Tris, pH 7.4. Radial absorbance distributions were recorded at 280 nm at 4 min intervals. Buffer densities were measured with a Mettler densimeter; viscosities were calculated from buffer composition using the program SEDNTERP [19], available from http://www.rasmb.bbri.org/. Sedimentation data were fit using a numerical solution of the Lamm equation implemented in the program SEDFIT [19], [20], available from www.analyticalultracentrifugation.com. The resulting c(s,ff_0_) distributions were converted to standard 20,w conditions using SEDNTERP [21].

## Results

**Figure 1:**
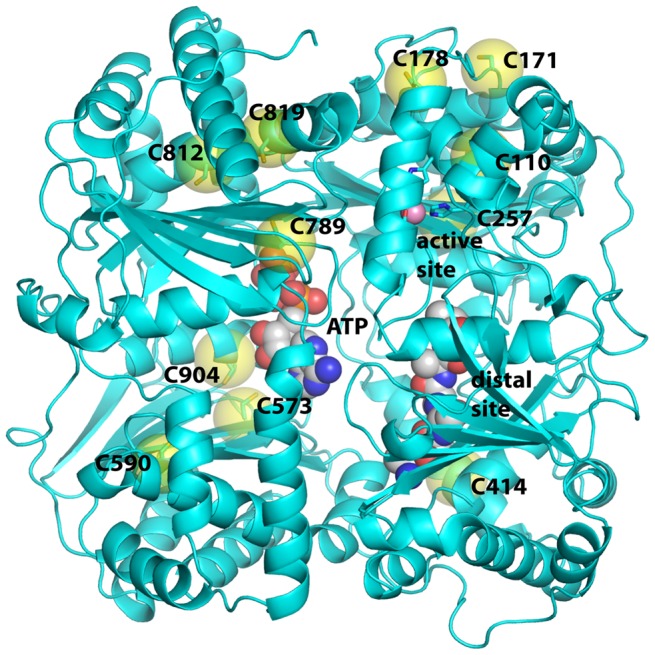
Ribbons representation of the IDE crystal structure. The position of cysteine residues, and other key features of IDE are indicated.

**Figure 2:**
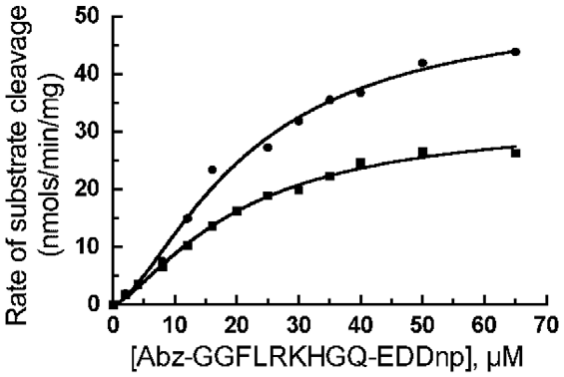
Comparison of the kinetics of IDE to cysteine-free IDE. Reactions of 200 µl contained 50 mM Tris-HCl buffer, pH 7.4, Abz-GGFLRKHGQ-EDDnp at the indicated concentrations and 1 µg of IDE (closed circles) or 2 µg of cysteine-free IDE (closed squares). The cleavage of the Abz fluorogenic substrate was determined as described in Methods. A higher amount of cysteine-free enzyme was used in order to achieve similar reaction rates between IDE and cysteine-free IDE. Shown are representative data from experiments that were conducted with two different enzyme preparations.

The rat IDE gene encodes a total of thirteen cysteine residues, however the start site for translation is usually at the second methionine in the sequence, which excludes the most N-terminally encoded cysteine. Thus for this study we only considered the 12 cysteine residues in the predominantly expressed enzyme form, **Figure 1**. When we generated a cysteine-free form of IDE by converting these 12 cysteine residues in rat IDE to serine we found an approximate 50% decrease in Vmax (51 µmols/min/mg for wild type IDE compared to 23 µmols/min/mg cysteine-free IDE) as well as a slight decrease in the Km for the substrate Abz–GGFLRKHGQ-EDDnp (26 µM vs. 19 µM for wild type IDE and cysteine-free IDE, respectively). There was essentially no change in the Hill coefficient, **Table 1** **and** **Figure 2**. This indicates that i) no particular cysteine residue is required for activity, a finding in agreement with those of others [12]–[15], [17], but that one or more cysteines are required for maximal activity and ii) a cysteine residue is not required for the substrate induced cooperativity characteristic of IDE with Abz-GGFLRKHGQ-EDDnp as substrate.

**Table 1:** Comparison of the kinetic properties of IDE and cysteine-free IDE with Abz-GGFLRKHGQ-EDDnp as substrate.

Enzyme form	Specific Activity (nmols/min/mg)	Hill Coefficient	Km (µM)
IDE	50.6±8	1.6±0.2	26.1±4
Cysteine-free IDE	28.3±4	1.8±0.3	18.6±2

Kinetic constants were determined by measuring rates at variable concentration of Abz-GGFLRKHGQ-EDDnp as substrate. Data were fit to the Hill equation.

When we examined the ability of a polyanion (ATP or TNP-ATP) to increase the rate of Abz-GGFLRKHGQ-EDDnp hydrolysis, we found that the cysteine-free enzyme was activated to a considerably lesser extent than the native enzyme. F**igure 3** shows that the cysteine-free IDE exhibits a maximal ∼20 fold activation by ATP or TNP-ATP compared to an ∼100 fold increase in activity with wild type IDE. In addition the K_A_ for ATP activation was ∼10 fold lower for the cysteine-free enzyme (0.06±0.01 mM) than for wild type IDE (0.69±0.06 mM), **Table 2**, while that for TNP-ATP was 0.06±0.01 µM for cysteine-free IDE compared to 0.64±0.07 µM for IDE. Thus although ATP binds to the cysteine-free enzyme as evidenced by its ability to increase the reaction rate, one or more cysteine residues are required to achieve maximal polyanion activation.

**Figure 3:**
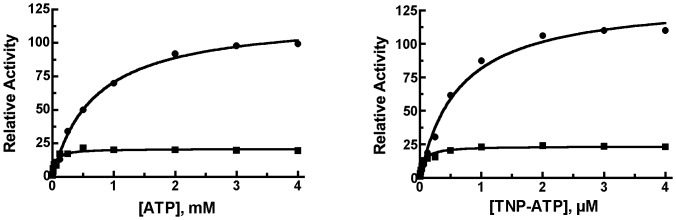
Comparison of the activation of IDE to cysteine-free IDE by ATP and TNP-ATP. Reactions of 200 µl contained 50 mM Tris-HCl buffer, pH 7.4, 10 µM Abz-GGFLRKHGQ-EDDnp and 1 µg of IDE (closed circles) or 2 µg cysteine-free IDE (closed squares) and the indicated amount of ATP (left) or TNP-ATP (right). Relative activity is defined as the activity observed at the indicated ATP or TNP concentration relative to the activity in their absence. A higher amount of cysteine-free enzyme was used as noted in Figure 2. Shown are representative data from experiments that were repeated twice using different enzyme preparations.

**Table 2:** Effect of converting individual cysteine residues to serine on IDE kinetics.

Enzyme form	Activity at 10 µM Abz substrate. (nmols/min/mg)	Fold Activation by 4 mM ATP	Fold Activation by 4 mM PPPi	K_A_ for ATP activation (µM)
IDE	8.4 +1.1	91±3	40±3	690±70
Cysteine-free	6.8+0.2	23±0.1	11.0±0.3	76±14
C110S	9.0±0.3	82±2	35±2	350±40
C171S	0.3±0.01	7±1	5±0.1	400±116
C178S	12.8±0/01	68±1	24±0.4	197±9
C257S	6.1±0.1	66±2	32±0.7	522±54
C414S	8.8±0.8	54±2	21±1.7	270±39
C573S	5.5±0.2	60±4	25±5.6	696±98
C590S	4.4±0.2	60±3	22±1.6	749±114
C789S	5.2±0.4	73±2	45±10	738±64
C812S	7.4±0.6	99±4	48±14	687±81
C819S	6.8±0.3	83±3	67±11	726±70
C904S	0.3±0.0	13±1	6±0.2	769±143
C966S	6.4±0.4	88±5	56±10	337±66
C904A	1.6±0.1	15±1	7±1.2	654±127

The activity of IDE and its cysteine mutants was determined using a fixed concentration of 10 µM Abz–GGFLRKHGQ-EDDnp as substrate. K_A_ was determined by varying [ATP] at a fixed substrate concentration of 10 µM. Data is expressed as the mean plus or minus the standard deviation. Each value represents measurements made two to four times.

In order to determine if a cystine might be present in IDE that affects its activity we tested the effect of the reducing agent Tris(2-carboxyethyl)phosphine (TCEP). We saw no affect of TCEP on activity or on the ability of ATP to activate the enzyme. As a control we tested the effect of TCEP on cysteine-free IDE and as expected did not observe a change in either activity or ATP activation.

In an attempt to identify the cysteine residue required for maximal activity and maximal polyanion activation, each of the 12 cysteine residues was individually converted to an isosteric serine residue. Although the majority of these mutants behaved similarly to wild type IDE in terms of their activity with 10 µM Abz-GGFLRKHGQ-EDDnp as substrate and their ability to be activated by ATP, **Table 2**, two mutants showed both a decreased maximal activity and a reduced activation by ATP; IDE-C171S and IDE-C904S. However, IDE-C171S expressed poorly and was difficult to purify. To rule out a specific effect of the serine residue we converted Cys171 and Cys904 to alanines. The IDE-C171A mutant expressed well and exhibited wild type enzyme activity and polyanion activation, so it was not further studied. However, substituting an alanine at position 904 produced an enzyme form that exhibited nearly the same properties as the cysteine-free enzyme, namely reduced activity and reduced activation by ATP, **Table 2**. IDE-C904A retained the allosteric properties of wild type IDE exhibiting a Hill coefficient of 1.5±0.2 with Abz-GGFLRKHGQ-EDDnp as substrate.

**Figure 4:**
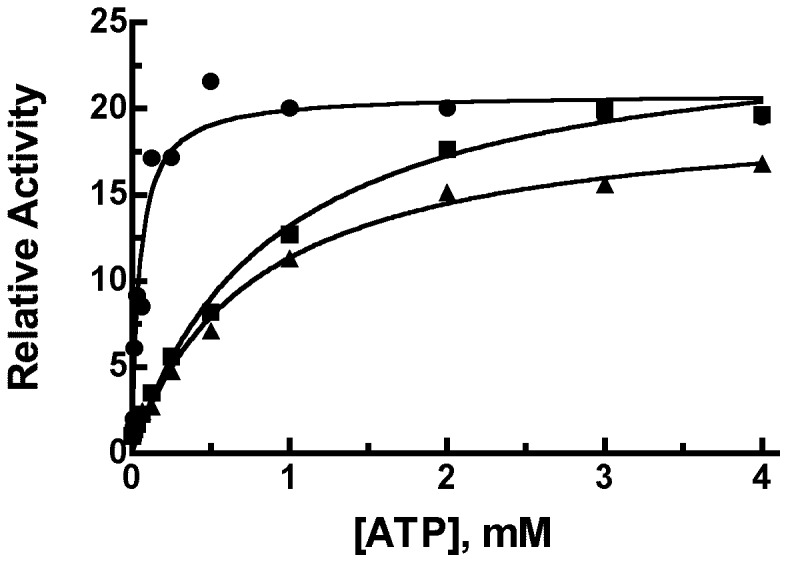
Comparison of the activation of IDE mutants by ATP. Reaction mixtures were as described in Figure 3 with ATP varied as indicated. Shown are cysteine-free IDE (2 µg - closed circles), IDE Cys904Ser (8 µg - closed squares), and IDE Cys904Ala (2 µg - closed triangles). The activity is plotted as the activity observed at the indicated ATP concentration relative to the activity in the absence of added ATP. Shown are representative data from experiments that were replicated with different enzyme preparations.

The K_A_ for ATP binding to IDE-C904A was the same as that obtained for wild type IDE, 0.65±0.13 mM. This is in contrast to the cysteine-free enzyme where the K_A_ for ATP deceased by ∼10 fold. Thus substitution of either Ala or Ser at position 904 produced an enzyme form that was a hybrid between the wild type enzyme and the cysteine-free enzyme. Relative to wild type IDE, IDE-C904A/S exhibits reduced catalytic activity and reduced activation by ATP, but the increase in ATP affinity seen for the cysteine-free enzyme is not observed in this variant, **Figure 4**. Thus a different serine or combination of serines must be responsible for increasing the affinity of the enzyme for ATP. We therefore measured ATP activation curves for each of the cysteine to serine mutants and found that no one mutant exhibited the low K_A_ seen for the cysteine-free enzyme, **Table 2**. As shown in **Table 2** several Cys to Ser mutations caused a modest decrease in the K_A_ for ATP, but not of the magnitude seen in cysteine-free IDE. These were C110 (K_A_ - 0.35 mM), C171 (K_A_ - 0.40 mM), C178 (K_A_ - 0.20 mM), C414 (K_A_ - 0.27 mM), and C966 (K_A_ - 0.34 mM). C110, C171, and C178 are clustered near the top of the domain 1 active site, while C414 is also near the distal part of the substrate binding site. C966 is in a disordered external loop not seen in the crystal structure. In order to determine if multiple cysteine residues could contribute to the binding of ATP we tested a previously generated mutant in which C110, C171, and C178 were converted to serines. This mutant showed a lower K_A_ for ATP (0.11 mM), than any of the individual mutations, suggesting that these and likely the other serines noted above produce small structural changes that together increase the affinity for ATP.

**Figure 5:**
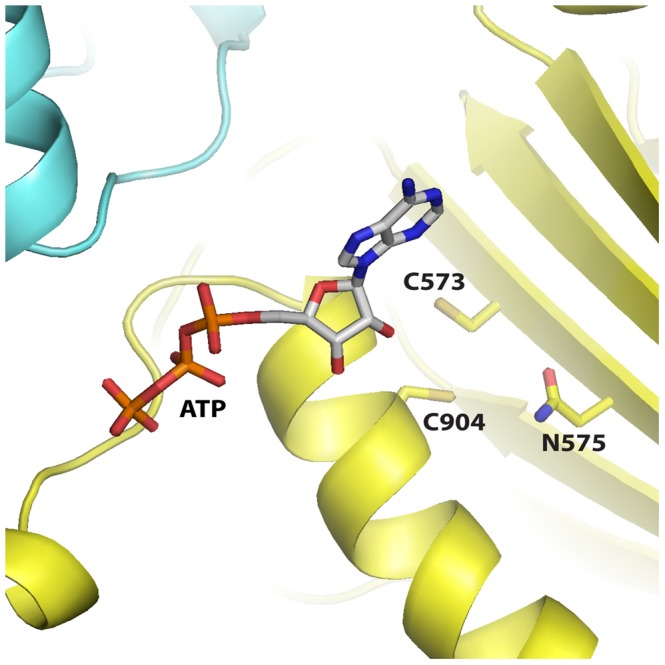
Structure of IDE showing the relative positions of Cys904, Cys573, and Asn575. The N- and C-terminal halves of the molecule are colored yellow and blue, respectively. Bound ATP is shown in stick representation.

**Figure 6:**
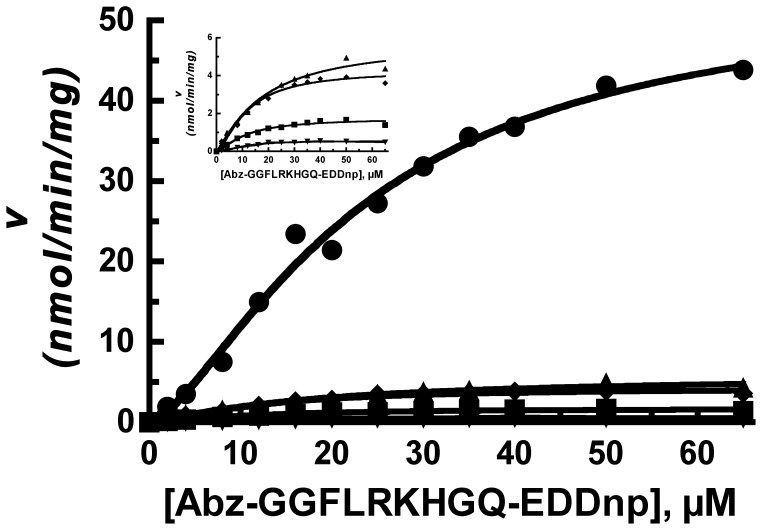
Comparison of the kinetics of IDE to C904S/A and to N575T/L. Reaction mixtures contained 50 mM Tris-HCl buffer, pH 7.4, 10 µM Abz-GGFLRKHGQ-EDDnp and either 1 µg of IDE (closed circles), 4 µg of IDE C904S (closed inverted triangle), 4 µg of IDE C904A (closed diamonds), 5 µg of IDE N575T (closed squares), or 2 µg of IDE N575L (closed triangles). The insert shows a comparison of the IDE mutants. Shown are representative data from experiments that were replicated with different enzyme preparations.

In order to understand the possible consequences of mutating C904 we examined its environment in the published x-ray structures of rat and human IDE [8], [10]. Although C904 is in position to form a disulfide bond with C573, this was ruled out as noted above by demonstrating an absence of any effect on activity when the enzyme was treated with the reducing agent TCEP. In addition mutating C573 to serine did not produce a significant effect. However, Cys904 is also in position to form a hydrogen bond with Asn575, **Figure 5**. We thus mutated Asn575 to Leu and Thr and looked at the effect of these mutations on activity. Interestingly both the N575T and N575L IDE mutants behaved similar to the C904S/A mutants. The Vmax for these mutants was decreased ∼7 to 30 fold relative to wild type IDE, **Figure 6**, while activation by ATP or TNP-ATP was reduced from the 100 fold seen with wild type IDE to ∼20 fold, similar to that seen in C904S/A. With the N575T and N575L mutants the K_A_ for ATP was essentially the same as for wild type IDE (∼0.6 mM), not the 10 fold lower value characteristic of the cysteine-free enzyme.

To extend this analysis we examined the activity of cysteine-free IDE with amyloid beta peptide 1–40 (Aß_1–40_) as a substrate. When cysteine-free IDE was incubated with Aß_1–40_ with the same amount of enzyme and for the same time, no detectable hydrolysis was observed (Figure S2, middle panel). Thus the amount of enzyme was increased to obtain a quantifiable rate (right panel Figure S2). This analysis showed that in contrast to the ∼2 fold reduction in activity seen with the fluorogenic peptide, the activity of cysteine-free IDE was decreased more than 20 fold with Aß_1–40_ as substrate, **Table 3**. This reduction in activity was recapitulated in the C904S/A mutants as well as with the N575T and N575L mutants, **Table 3**. As a control we tested two other cysteine to serine mutations that had little effect on the activity towards the fluorogenic substrate, C812S and C819S. As seen with the fluorogenic substrate these control mutations exhibited activity that was similar to the wild type enzyme.

**Table 3:** Activity of IDE and its mutants with amyloid beta peptide and insulin as substrates.

Enzyme form	Activity with Aß_1–40_ as substrate (nmols/min/mg)	Activity with insulin as substrate (nmols/min/mg)
IDE	881.0±25.6	12.0±2.8
Cysteine-free IDE	16.3+2.9	2.5±0.5
C904S	4.1±0.1	<0.1
C904A	26.7±3.7	1.0±0.3
N575T	6.8±1.7	2.8±0.7
N575L	31.6±16.6	2.8±0.6
C812S	794±14	5.8±0.7
C819S	990±76	8.6±0.7

Reactions containing 10 µM Aß_1–40_ or 10 µM insulin as substrates in 50 mM Tris-HCl buffer, pH 7.4, were incubated at 37°C for 30 to 60 min. Rates were determined by measuring the disappearance of the substrate peak by HPLC as described in Methods. IDE^C812S^ and IDE^C819S^ were used as controls to demonstrate the specificity of effects resulting from mutating Cys 904 and Asn 575. Data is expressed as the mean plus or minus the standard deviation. Each value represents measurements made two to five times.

This study was then extended to include insulin as a substrate. Here again when tested with the same amount of enzyme and for the same time, cysteine free IDE showed little hydrolysis IDE, (Figure S2, lower middle panel). As with Aß_1–40_ as substrate amount of cysteine-free IDE enzyme was increased to obtain a quantifiable rate (lower right panel Figure S2). This analysis showed that cysteine-free enzyme exhibited ∼20% of wild type IDE activity towards insulin, **Table 3**. As with the other substrates tested the single mutation of C904S/A also produced a large decrease in insulin degrading activity as did the N575T and N575L mutants. A relatively small effect (30 to 50% reduction in activity) was seen with the control mutations C812S and C819S, in contrast to the 80% to greater than 99% reduction in activity seen with the Cys904 or Asn575 mutations.

**Figure 7:**
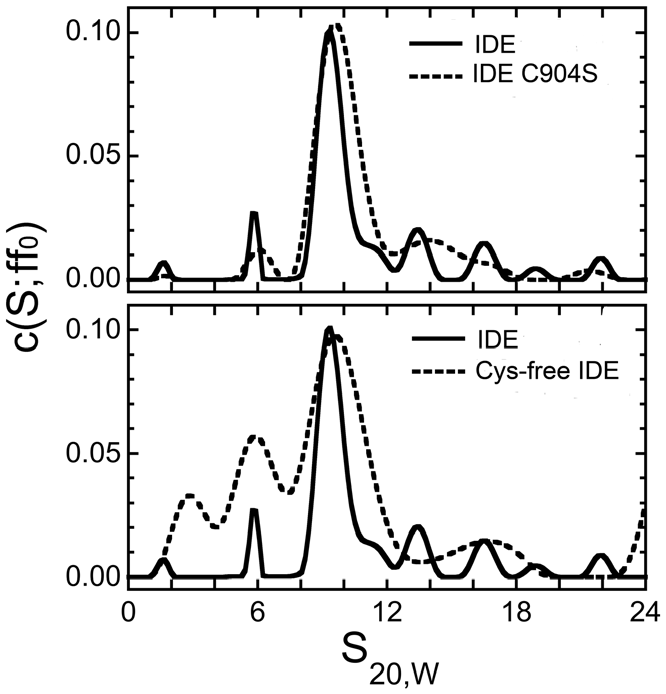
Sedimentation velocity distributions of wild type and mutant IDEs. Sedimentation velocity measurements were made at 4°C and 40,000 rpm. The c(s, ff_0_) distributions calculated with SEDFIT, have been corrected to 20,w conditions. This analysis was repeated with at least two different enzyme preparations. In order to further validate the sedimentation pattern the enzyme preparations were resubjected to gel filtration prior to repeating the analysis.

We next determined the oligomerization state of IDE, cysteine-free IDE, and the IDE C904S mutant by sedimentation equilibrium. As shown in **Figure 7**, wild type IDE showed primarily the dimeric (9.3 s) form with small amounts of monomer (5.8 s) and higher oligomers present. This pattern did not change when the enzyme was treated with TCEP or the polyanion activator PPPi, data not shown. Cysteine-free IDE shows an increase in monomer content, but this change cannot account for the large differences in activation relative to the wild type enzyme. A smaller species (2.3 s) is also observed in the cysteine-free preparation, and may represent a contaminant or breakdown product. The C904S mutant, which has similar activity to the cysteine-free iDE, does not exhibit the increase in monomer content or the small contaminant. Both the cysteine-free and Cysteine904Serine mutants show broadening of the dimer and oligomer peaks suggesting a more rapid equilibrium between monomer - dimer and higher oligomers.

## Discussion

In this study we have examined the role of cysteine residues in the activity and polyanion activation of IDE. We have shown that although cysteine residues are not required for IDE function, cysteine 904 is required to achieve maximal activity. The importance of cysteine residues for maximal activity varies with different substrates. With the small synthetic peptide Abz-GGFLRKHGQ-EDDnp the conversion of cysteines to serines decreases activity by about 2 fold, with insulin as substrate activity is decreased five fold, while with Aß as substrate activity decreases more than 20 fold. As noted in the introduction, there have been a number of studies that utilized the cysteine-free form of IDE. The results reported here suggest that the results obtained in those studies must be interpreted cautiously, since this form of IDE does not retain full catalytic activity.

In addition to the decrease in activity noted above, conversion of cysteines to serines causes a ∼10-fold decrease in the ability of polyanions to activate the enzyme with Abz-GGFLRKHGQ-EDDnp as substrate. Again mutation of cysteine 904 was primarily responsible for this effect. Substitution of serines for all of the cysteines increases the affinity of the enzyme for ATP. Substitution of Cys904 alone does not recapitulate this affect, rather a group of cysteines acting cooperatively appear to produce the decreased K_A_ for ATP. Since the group of cysteines that contribute to this effect are more than 25 Å from the polyanion binding site, this suggests that conformational differences contribute to the increased affinity for ATP, rather than direct interactions with the polyanion.

Crystal structures of IDE show a possible hydrogen bond between cysteine 904 and asparagine 575. The finding that mutation of asparagine 575 to leucine or threonine mimicked the effect of mutating cysteine 904 suggests that cysteine 904 and asparagine 575 do indeed interact, likely through hydrogen bonding. Less likely is that both residues play independent roles in IDE activity. The loss of this hydrogen bond most likely alters the conformation of the enzyme such that activity and polyanion activation are suppressed, but not eliminated. In comparing the structures of IDE [11] and cysteine-free IDE [13] no obvious conformational differences are apparent. However, such conformation differences in the cysteine-free enzyme may be unfavorable in the crystal structure.

What we do see is a change in the distribution between monomers and dimers between IDE, which is primarily dimeric, and the cysteine-free form of IDE, which has a higher proportion of monomer. This change in oligomeric state does not correlate with changes in IDE activity since as much as a 20 fold decrease in activity is seen with Aß as substrate with only an ∼2 fold decrease in dimer content. Additionally the C904S mutant is primarily a dimer like wild type IDE, yet differs significantly in activity from wild type IDE.

The data is consistent with a model in which disruption of the interaction between cysteine 904 and asparagine 575 results in a small conformational change between dimeric units. We hypothesize that the conformation of the mutant dimer differs from that of the wild type dimer and is inherently less active and less able to be activated by polyanions.

## Supporting Information

Figure S1 **Analysis of IDE purity by SDS-PAGE.** IDE and its mutant forms were purified as described in Materials and Methods and 2 µg of protein was analyzed by SDS-PAGE using Coomassie staining to visualize the protein bands. Panel A. Wild Type IDE, Panel B. Cysteine free IDE, Panel C. IDE-C904S. Molecular weight standards are included the first lane in each panel.
(TIF)

Figure S2 **HPLC chromatograms showing the hydrolysis of Aß_1–40_ and insulin by wild type IDE and cysteine-free IDE used to calculate reaction rates.** Top. Hydrolysis of Aß_1–40_. Reaction mixtures containing 10 µM Aß_1–40_ in 50 mM Tris-HCl, pH 7.4, were incubated with the indicated amounts wild type IDE (30 min incubation) or cysteine-free IDE (middle panel 30 min) or right panel 60 min). Samples were then subjected to HPLC as described in Materials and Methods and the activity was calculated on the basis of the difference in peak height between Aß_1–40_ alone (curve A) or Aß_1–40_ in the presence of enzyme (curve B). Bottom, Hydrolysis insulin. Reaction mixtures as above containing 10 µM insulin were reacted with the indicated amounts of IDE or cysteine-free IDE for 60 min. Samples were analyzed as noted above with activity was calculated on the basis of the difference in peak height between insulin alone (curve A) or insulin in the presence of enzyme (curve B).
(TIF)
