# Supplementary figures and images for: Cysteine 904 Is Required for Maximal Insulin Degrading Enzyme Activity and Polyanion Activation
Source: PLoS One. 2012 Oct 15;7(10):e46790. doi: 10.1371/journal.pone.0046790 (PMC3471918; doi:10.1371/journal.pone.0046790)

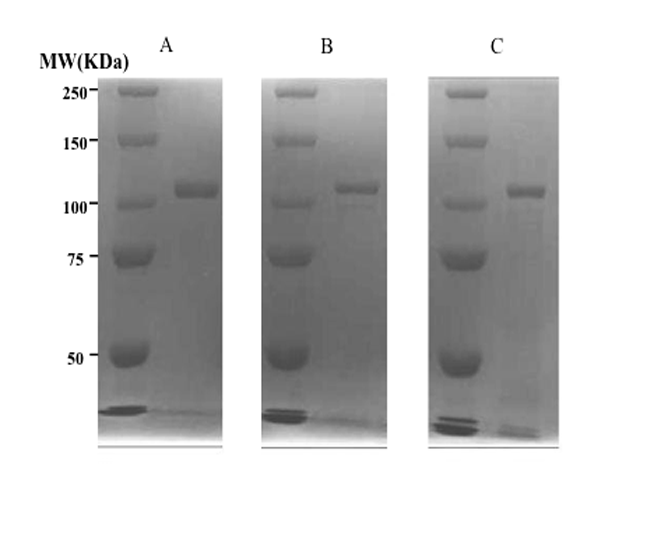

Supplement: Figure S1 — Analysis of IDE purity by SDS-PAGE. IDE and its mutant forms were purified as described in Materials and Methods and 2 µg of protein was analyzed by SDS-PAGE using Coomassie staining to visualize the protein bands. Panel A. Wild Type IDE, Panel B. Cysteine free IDE, Panel C. IDE-C904S. Molecular weight standards are included the first lane in each panel. (TIF) [file pone.0046790.s001.tif]

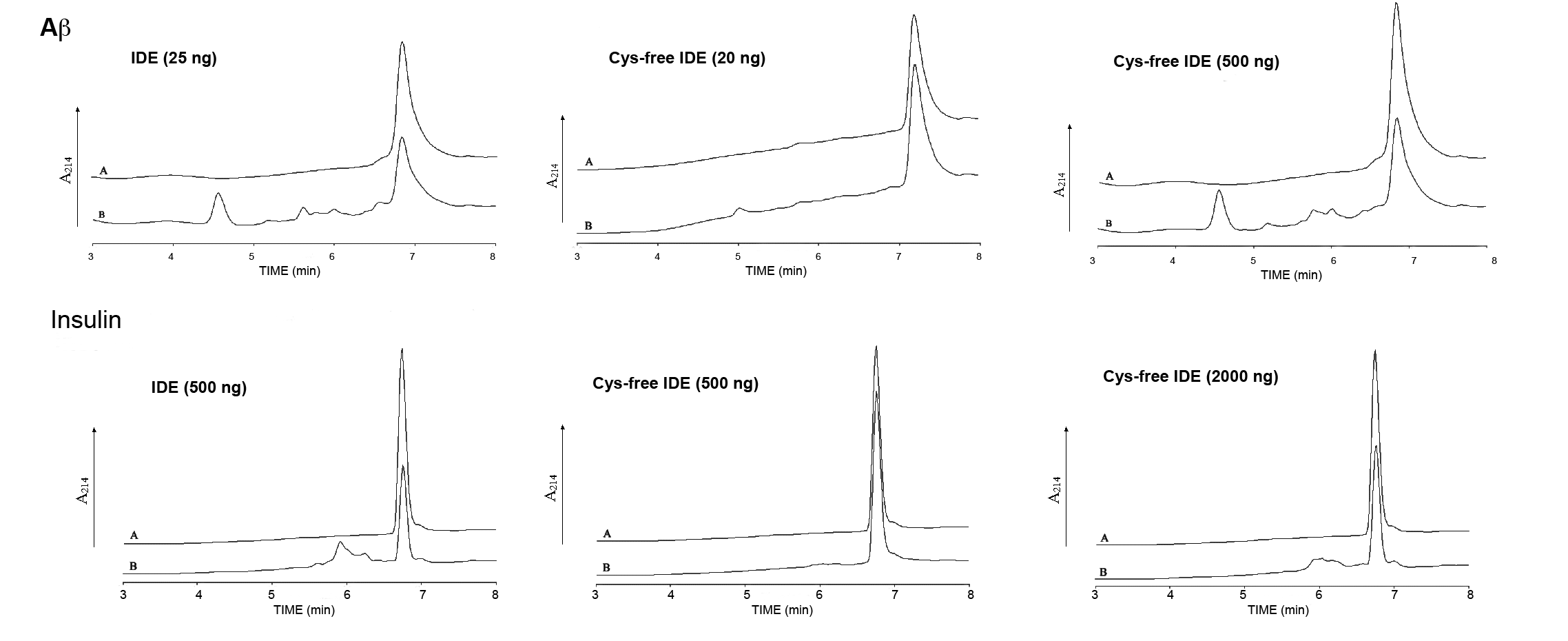

Supplement: Figure S2 — HPLC chromatograms showing the hydrolysis of Aß1–40 and insulin by wild type IDE and cysteine-free IDE used to calculate reaction rates. Top. Hydrolysis of Aß1–40. Reaction mixtures containing 10 µM Aß1–40 in 50 mM Tris-HCl, pH 7.4, were incubated with the indicated amounts wild type IDE (30 min incubation) or cysteine-free IDE (middle panel 30 min) or right panel 60 min). Samples were then subjected to HPLC as described in Materials and Methods and the activity was calculated on the basis of the difference in peak height between Aß1–40 alone (curve A) or Aß1–40 in the presence of enzyme (curve B). Bottom, Hydrolysis insulin. Reaction mixtures as above containing 10 µM insulin were reacted with the indicated amounts of IDE or cysteine-free IDE for 60 min. Samples were analyzed as noted above with activity was calculated on the basis of the difference in peak height between insulin alone (curve A) or insulin in the presence of enzyme (curve B). (TIF) [file pone.0046790.s002.tif]
